# Polymeric Nanocomposites of Boron Nitride Nanosheets for Enhanced Directional or Isotropic Thermal Transport Performance

**DOI:** 10.3390/nano14151259

**Published:** 2024-07-27

**Authors:** Buta Singh, Jinchen Han, Mohammed J. Meziani, Li Cao, Subhadra Yerra, Jordan Collins, Simran Dumra, Ya-Ping Sun

**Affiliations:** 1 Department of Chemistry, Clemson University, Clemson, SC 29634, USAsdumra@g.clemson.edu (S.D.); 2 Department of Chemical and Materials Engineering, University of Dayton, Dayton, OH 45469, USA; 3 Department of Natural Sciences, Northwest Missouri State University, Maryville, MO 64468, USA

**Keywords:** boron nitride nanosheets, polymeric composites, in-plane thermal conductivity, cross-plane thermal conductivity

## Abstract

Polymeric composites with boron nitride nanosheets (BNNs), which are thermally conductive yet electrically insulating, have been pursued for a variety of technological applications, especially those for thermal management in electronic devices and systems. Highlighted in this review are recent advances in the effort to improve in-plane thermal transport performance in polymer/BNNs composites and also the growing research activities aimed at composites of enhanced cross-plane or isotropic thermal conductivity, for which various filler alignment strategies during composite fabrication have been explored. Also highlighted and discussed are some significant challenges and major opportunities for further advances in the development of thermally conductive composite materials and their mechanistic understandings.

## 1. Introduction

**Figure 1:**
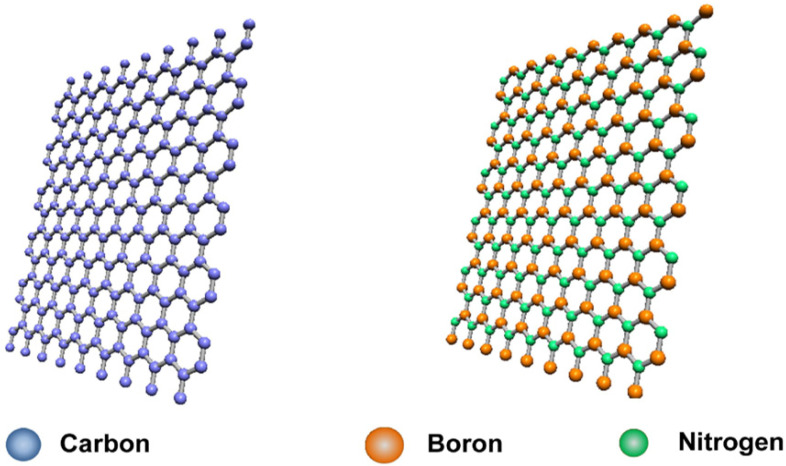
Graphene (**left**) and boron nitride nanosheet (**right**). Reproduced/Adapted with permission from Ref. [19].

Thermal management has become a critical challenge in modern electronic devices, including some high-performance batteries that generate heat but become hazardous with the rising temperature, and many increasingly popular cellular and radiofrequency devices that are characterized by continuous miniaturization, improved functionality, and higher power density [1,2,3,4,5,6]. For example, currently, overheating in electronics is the major cause of device failures and reductions in their work performance and lifetime [7,8]. Therefore, there has been an urgent quest for the development of effective thermal management technology derived from materials of superior thermal transport characteristics for improving the thermal performance, life cycle, reliability, and safety of devices and related systems [9,10,11,12,13,14,15,16,17]. In this regard, materials of high directional or isotropic thermal conductivity are particularly valuable. Among those attracting much recent attention have been polymeric composites with nanoscale fillers of high thermal conductivity (TC), especially 2D nanofillers of graphene nanosheets (GNs, Figure 1 (left)) [14,18] and boron nitride nanosheets (BNNs, Figure 1 (right)) [3,19]. GNs are high in both TC and electric conductivity (EC), so are their polymeric composites, but BNNs are high in TC but electrically insulating, which represents a unique advantage for applications in electronic devices and systems [3,17,19,20,21,22,23,24,25,26,27,28,29,30,31,32,33,34].

Theoretically estimated TC values of BNNs are up to 2000 Wm^−1^K^−1^ in-plane and up to 30 Wm^−1^K^−1^ cross-plane [31,35,36,37,38,39]. Their other unique and/or useful properties in addition to being electrically insulating include their extreme thermal stability and chemical resistance against oxidation at temperatures up to 1000 °C and their passivity to reactions with acids and melts [36,40,41,42,43], thus being especially suitable for uses under extreme conditions [36,43]. BNNs for polymeric composites are typically obtained from the exfoliation processing of hexagonal boron nitride (h-BN), a relatively abundant and inexpensive precursor, so that polymeric composites of high TC performance may be produced at low costs. All of these may have been driving the increasingly active recent development efforts on high-performance and light-weight polymer/BNNs composite materials for thermal management applications [3,19,34].

**Figure 2:**
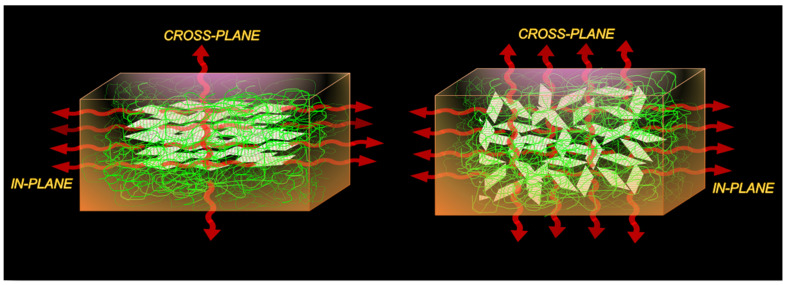
A schematic illustration for in-plane and cross-plane thermal conduction in polymer/BNNs nanocomposites.

This review is designed to highlight the significant recent advances, focusing on new progress made since our last account in 2021 [19], in the development of polymeric composites with BNNs for high directional or isotropic TC performance (Figure 2). In our previous accounts [3,19], we emphasized the efficient exfoliation techniques for high-quality BNNs, the understanding of their characteristic properties, and the performance of their thermally conductive nanocomposite derived materials along with their challenges. The major opportunities with these materials for their technological applications and some of their critical challenges are also identified and discussed.

## 2. BNNs for Thermally Conductive Polymeric Nanocomposites

For its layered structure and being isoelectronic to graphite, h-BN is often nicknamed “white graphite,” and by extension, BNNs are considered as being analogous to few-layer GNs with some additional important property advantages. Thus, it seems logical to disperse BNNs into polymeric matrices for nanocomposite materials of high TC but no EC at all. Indeed, the significant progress made in the development of polymer/BNNs composites for the desired thermal transport and other properties has demonstrated great promise. However, h-BN is meaningfully different from graphite, as are BNNs from GNs. The inter-layer forces in h-BN are ionic interactions in nature, stronger than the van der Waals forces in graphite, which makes the exfoliation of h-BN into BNNs more difficult. A consequence of the more difficult exfoliation, for which some of the commonly employed processing methods may actually be considered as a combination of exfoliation and hammering or breaking, is that the resulting BNNs are not as well defined as their carbon counterparts in terms of the targeted planar structure or morphology, which may significantly affect their composition with polymers and/or their dispersion in the resulting polymer composites. BNNs are also much harder materials than their carbon counterparts, less compatible with softer polymer matrices, and their alignment in polymeric matrices with external forces is more challenging. These characteristic features of BNNs should be taken into consideration in their uses as nano-fillers in polymer matrices for composites of high thermal transport performance [20,44]. For example, for higher in-plane TC in polymer composites of planar nano-fillers, the filler alignment with strategies such as mechanical stretching is generally less effective for BNNs than their carbon counterparts, often requiring special efforts [3,19,30,32,34,37,45,46,47,48,49,50,51].

In principle, BNNs could be prepared “bottom-up” [34,52,53,54,55,56] with processing methods such as chemical vapor deposition (CVD) [55,56] or even by conversion from GNs with boron and nitrogen sources at high temperatures to exploit the higher stability of BNNs than that of GNs, but in reality, BNNs in the quantity required for polymer composites have been prepared by exfoliation of h-BN. Among the commonly employed exfoliation methods are ultrasonication in selected solvents [3,27,46,56,57,58,59,60,61,62], ball milling aided by organic dispersion agents [19,51,58,63,64,65,66,67,68,69,70,71,72,73,74,75,76,77], strong acids and bases [19,58,63,78], supercritical fluids, and ionic liquid processing [19,58,63,79,80,81]. In our last review [19], the advantages and disadvantages of these exfoliation methods were evaluated, for which the critiques are still valid. A few other methods combining ultra-low temperature (liquid nitrogen) treatment of h-BN with subsequent microwave processing or ultrasonication have been tested more recently with some promising results [82,83].

## 3. Polymer/BNNs Composites of Enhanced In-Plane TC

Since BNNs are nano-fillers of a more planar structure, they are more popular in polymer composites of high in-plane thermal transport performance measured by thermal conductivity (TC or k) values obtained directly or more often derived from experimentally determined thermal diffusivity (TD or α) values. These values are determined by using measured or estimated densities (*ρ*) and heat capacities (*C*_p_) of the materials under consideration [84,85,86] as follows:

TC = *ρC*_p_TD (1)

Among the recently reported in-plane TD values (Table 1), the overwhelming majority were measured using NETZSCH laser flash apparatuses (LFAs) (Taufkirchen, Germany) [87], such as LFA-447 NanoFlash (Taufkirchen, Germany) and LFA-HyperFlash instruments (Taufkirchen, Germany). The other measurement methods included the Hot Disk Transient Plane Source (TPS) (Hot Disk AB, Gothenburg, Sweden) [88], the Ulvac LaserPIT instrument (Chigasaki, Kanagawa, Japan) based on the modified Angstrom’s method designed specifically for measuring in-plane TD values of thin films [89], and the steady-state method measuring TC directly [90]. In a previous review [19], a puzzling phenomenon was identified such that the handful of in-plane TC values obtained directly by the steady-state method were mostly considerably higher than those from measurements by other methods. For a clearer understanding of such a phenomenon and also for the general comparability of different measurement methods, it was proposed that some absolute calibrations with standard specimens of known TD/TC values for the individual methods and/or comparative calibrations across the different methods should be performed carefully and systematically to establish the absolute and relative accuracies of the reported TD/TC values and their corresponding measurement methods. Such calibrations are obviously critical to the healthy advances of the entire research field. Here is the same proposal again in the absence of any such efforts since the last review.

For in-plane TC, polymer/BNNs composite films are commonly used. Their fabrication is often by solution casting or vacuum-assisted filtration methods for aligning BNNs in the films, preferred due to their simplicity and the ability to control the film thickness [3,19,50,51,64,127,128,129]. Among their disadvantages is the more difficult control of some film properties, such as the potential aggregation of fillers and possible voids between fillers and the polymer matrix. Nevertheless, these methods and their variations have remained popular.

**Figure 3:**
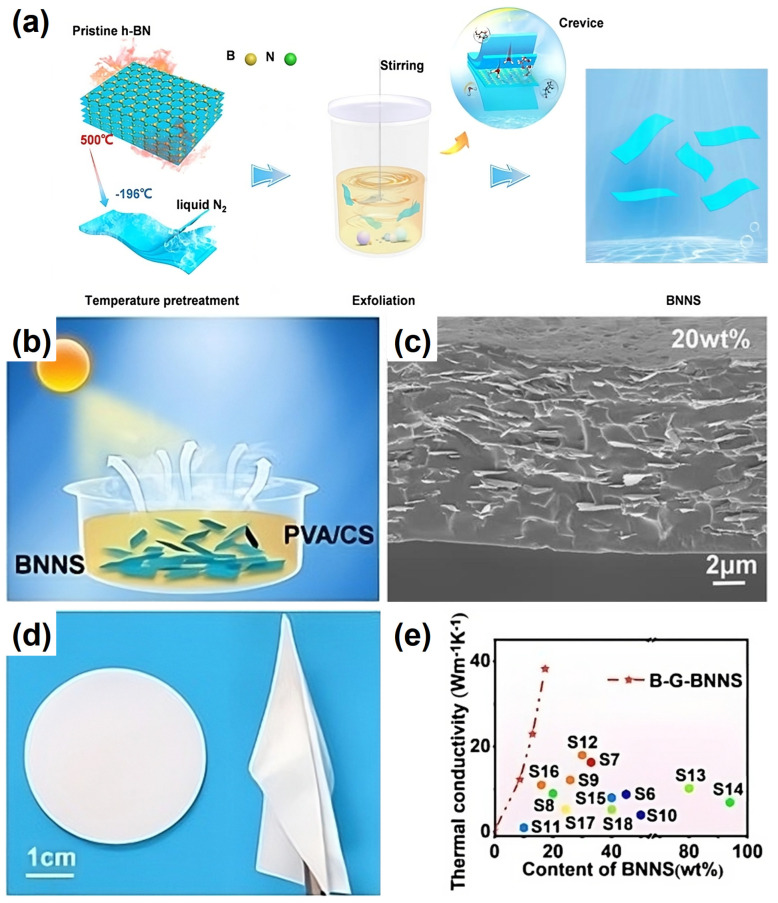
(**a**) Schematic illustration of the exfoliation and functionalization process. (**b**) The fabrication process of BNNs/PVA/CS composite films. (**c**) Cross sectional SEM picture of B-G-BNNs/PVA/CS with a filler content of 20 wt%. (**d**) Photograph of the film samples. (**e**) The films demonstrate various thermal conductivity with a different filler content of B-G-BNNs, and the in-plane thermal conductivity of B-G-BNNs/PVA/CS films was compared with that of other representatives’ works. Reproduced/Adapted with permission from Ref. [93].

Li, et al. [93] prepared a viscous dispersion of BNNs with poly(vinyl alcohol) (PVA) and chitosan (CS) and used a blade for the casting of (PVA + CS)/BNNs composite films. The BNNs were obtained from the exfoliation process involving the thermal treatment of h-BN at 500 °C and then rapid cooling in liquid nitrogen, and subsequently ball milling. The composite film with a BNNs content of 20 wt% exhibited an in-plane TC of 38.21 Wm^−1^K^−1^ (Figure 3). Chen, et al. [96] exfoliated h-BN in a suspension with carboxymethylated cellulose nanofibrils (CCNF) by using a probe sonicator and then used the mixture for the fabrication of CCNF/BNNs composite films that are water resistant. The in-plane TC value of the film with 50 wt% BNNs loading was 17.31 Wm^−1^K^−1^.

Han, et al. [98] applied a solvent-free cyclic layer-by-layer blade-casting method to the preparation of epoxy resin composite films with BNNs subjected to functionalization by ionic liquid. At 45 wt% loading of BNNs, the composite film exhibited an in-plane TC of 8.3 Wm^−1^K^−1^ and a much lower cross-plane TC of 0.8 Wm^−1^K^−1^. A similar layer-by-layer approach was employed by Nie, et al. [109] for the preparation of PVA/BNNs composites. The BNNs were obtained from ball milling of h-BN in the presence of a coordination polymer that would melt to produce ionic fragments under the ball-milling conditions, with the expectation for the fragments to insert into h-BN inter-layers for enhanced exfoliation. For the composite with 30 wt% BNNs loading, its in-plane TC was 11.5 Wm^−1^K^−1^.

Yan, et al. [102] employed vacuum-assisted filtration to prepare PVA/BNNs composite films using BNNs obtained from the exfoliation of h-BN with a microfluidic technique. The in-plane TC of the composite with 83 wt% BNNs loading was 67.6 Wm^−1^K^−1^. In a similar fabrication, Yang, et al. [101] prepared PVA/BNNs composite films, for which the BNNs were from the exfoliation of functionalized h-BN through the etching and wedging action of dehydrated boric acid at high temperature and subsequent ball milling. The in-plane TC values of the films were up to 9.2 Wm^−1^K^−1^ and 27.3 Wm^−1^K^−1^ at ∼60 wt% and ∼90 wt% BNNs loading, respectively, but the cross-plane TC values were much lower, less than 1.1 Wm^−1^K^−1^ even at the high BNNs loading of ∼90 wt%.

**Figure 4:**
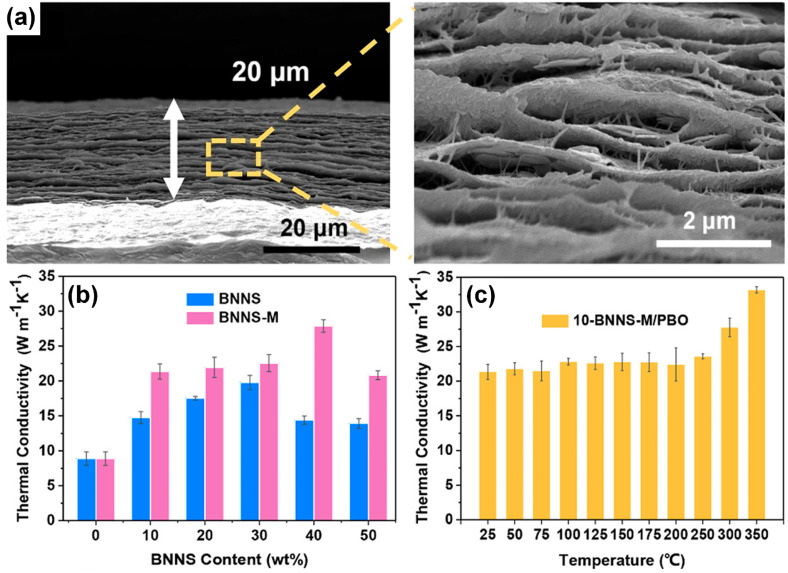
(**a**) Cross-sectional view of 10-BNNs-M/PBO paper by SEM. (**b**) Comparison of the in-plane thermal conductivities of the BNNs-M/PBO paper and the BNNs/PBO paper with different filler content. (**c**) In-plane thermal conductivity of 10-BNNs-M/PBO paper at different temperatures. Reproduced/Adapted with permission from Ref. [105].

There were efforts to incorporate BNNs into nanofibers, such as aramid nanofibers [106] or cellulose nanofibers coupled with carbon nanotubes [34,107], for composite films. The in-plane TC values of the films were pretty good, up to 28.75 Wm^−1^K^−1^ at 70 wt% BNNs loading [106], but hardly exceptional. However, the use of nanofibers could have other benefits. For example, Chen, et al. [105] used poly-*p*-phenylene benzobisoxazole nanofibers as matrix materials for their ultra-strong mechanical properties, high TC (8.89 Wm^−1^K^−1^), and excellent thermal stability, even though their composites with BNNs exhibited similarly good but not exceptional in-plane TC values, 21.34 Wm^−1^K^−1^ and 27.87 Wm^−1^K^−1^ at BNNs loadings of 10 wt% and 40 wt%, respectively (Figure 4). Interestingly, Sun, et al. [94] reported that when polydopamine-functionalized BNNs were used with nanofibers for composite films, the in-plane TC value could reach 45.15 Wm^−1^K^−1^ at 37.5 wt% loading of the functionalized BNNs.

Hot pressing is another fabrication method considered as being advantageous for creating a dense interconnected network of BNNs and driving their in-plane orientation in the composite films [64,128,130,131]. In a typical protocol, the composite is placed in a mold, heated at high temperature, and compressed under high pressure to the desired shape, followed by cooling back to room temperature. Liu, et al. [97] applied the method by first preparing microspheres of BNNs dispersed in a phthalonitrile resin containing a curing agent, with the BNNs obtained from ball milling h-BNs, and then hot pressing and post curing. The in-plane and cross-plane TC values of the composite film at 40 wt% BNNs loading were 7.84 Wm^−1^K^−1^ and 1.62 Wm^−1^K^−1^, respectively. Similar hot pressing was used by Gou, et al. [100] to prepare poly(vinylidene fluoride-*co*-hexafluoropropylene)/BNNs composite films, which at 25% BNNs loading exhibited an in-plane TC of 7.26 Wm^−1^K^−1^. In another study of hot pressing with a variation, Du, et al. [104] first spray coated a dispersion of BNNs onto a polyurethane web film, followed by annealing above the melting point of the polymer and hot pressing for the polyurethane/BNNs composite film. The protocol was aimed at making the BNNs well aligned and tightly packed along the in-plane direction. For the films of 20 wt% and 50 wt% BNNs content, the in-plane TC values were 6.36 Wm^−1^K^−1^ and 20.65 Wm^−1^K^−1^, respectively, and interestingly, the cross-plane TC values were relatively high as well (2.51 Wm^−1^K^−1^ and 5.77 Wm^−1^K^−1^, respectively).

Zhou, et al. [108] used the hot pressing method for more flexible composite films. In the preparation, BNNs were obtained from the sonication exfoliation of h-BN with γ-methacryloxy propyl trimethoxyl silane, followed by their melt blending with polypropylene at a high temperature and then folded many times with hot pressing. The composite film folded 20 times with a 19.1 vol% BNNs content exhibited an in-plane TC of 4.08 Wm^−1^K^−1^. Similarly in the study by Wang, et al. [95], BNNs containing OH groups were incorporated into a self-healing elastomer matrix for hot pressing into the composite films. It was found that the in-plane and cross-plane TC values of the films were film thickness dependent, higher in thinner films, reaching up to 12.62 Wm^−1^K^−1^ and 0.63 Wm^−1^K^−1^, respectively, at a thickness of about 75 microns of a film containing 38 wt% of BNNs.

**Figure 5:**
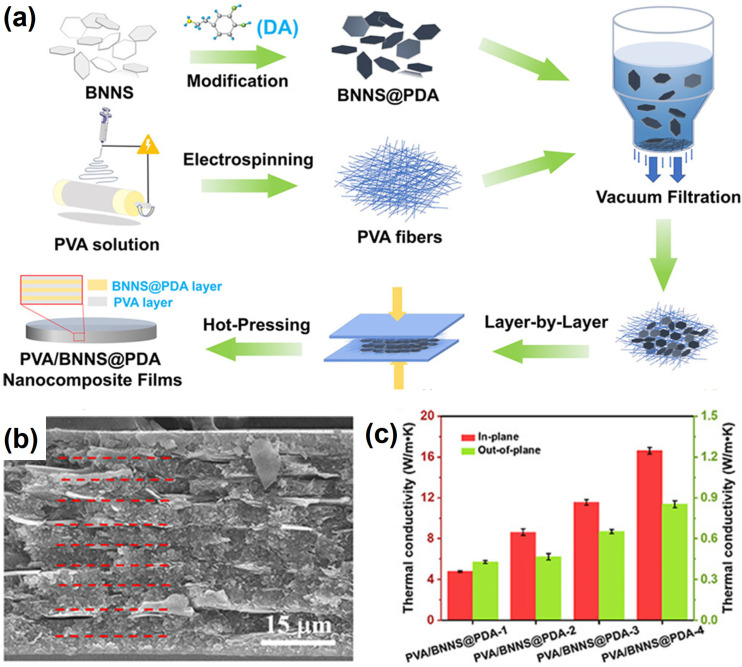
(**a**) Schematic diagram of the fabrication process of PVA/BNNs@PDA nanocomposites. (**b**) SEM cross-section images of PVA/BNNs@PDA-4 at different magnifications. The red dashed lines indicating the alternate layer interface. (**c**) In-plane and out-of-plane thermal conductivities of the PVA/BNNs@PDA nanocomposites with different BNNs@PDA contents. Reproduced/Adapted with permission from Ref. [103].

Electrospinning is based on the use of a high voltage to produce a charged jet from a polymer/filler solution, followed by stretching and elongation upon volatilization of the solvent to generate nanofibers of various diameters depending on the processing parameters. It has been employed as a fabrication technique for highly aligned composite fibers in which BNNs are similarly aligned. For example, Zhou, et al. [103] used electrospinning to produce PVA nanofiber mats and then deposited the polydopamine (PDA)-modified BNNs onto the mats with vacuum filtration. The resulting composite nanofiber mats were stacked and then packed by hot pressing to form composite films of PVA with PDA-modified BNNs. The film with 35.54 wt% loading of PDA-modified BNNs exhibited in-plane and cross-plane TC values of 16.6 Wm^−1^K^−1^ and 0.85 Wm^−1^K^−1^, respectively (Figure 5). Wang, et al. [110] coupled electrospinning with electrospraying to take advantage of their similar operating conditions for the fabrication of polyacrylonitrile/BNNs composite films. The preparation of the precursor composite nanofibers and the layer-by-layer processing were largely similar to those of Zhou, et al. [103] described above, except that eletrospraying was used for the dispersion of the BNNs. The in-plane TC value of the composite film at 40 wt% BNNs loading was up to 24.98 Wm^−1^K^−1^. Yu, et al. [111] used a similar electrospinning–electrospraying combination for the preparation of polyimide/BNNs composite films, but the in-plane TC value was only 7.58 Wm^−1^K^−1^ at 50 wt% filler loading.

Wang, et al. [126] developed a strategy of coupling cross-linking in composite films with mechanical stretching for filler orientation to in-plane TC performance. The strategy was adopted by Tu, et al. [92] in the preparation of strong and high TC cellulose/BNNs composite films. In the preparation, epichlorohydrin was used for the cross-linking. The gel-like cellulose/BNNs composites were stretched to three times their original length for the filler alignment, followed by the immobilization treatment and air-drying. The resulting composite film at 50 wt% BNNs loading exhibited in-plane and cross-plane TC values of 20.41 Wm^−1^K^−1^ and 0.69 Wm^−1^K^−1^, respectively.

The recent results highlighted above and obtained in other studies, some of which are summarized in Table 1, have further confirmed that the performance of the nanocomposites is subject to many effects, such as the fabrication methods and conditions of BN exfoliation and nanocomposite processing, loading, TC/TD measurement techniques and limitations, and various other complications. For the desired performance enhancement, the progress has generally been slow and mostly incremental since our last account in 2021 [19], with only a few benchmark in-plane TC values marked in bold in Table 1. For example, the use of functionalized BNNs with a high aspect ratio and their integration with chitosan or polydopamine PDA/poly-p-phenylene benzodiazole (PBO) as dispersing agents for composite films [93,94] improved the interfacial compatibility and adhesion and reduced phonon scattering, resulting in more thermally conductive nanocomposite films, with observed in-plane TC values up to ~38.21 and 45.15 Wm^−1^K^−1^ at BNNs loading of 20 and 37.5 wt%, respectively. These results have collectively established that the preparation of high-quality BNNs of fewer layers and larger aspect ratios, consistent with the theoretical rationale on the longer phonon mean free paths and reduced phonon scattering, and the achievement of good interface compatibility and good dispersion into polymer matrices, are prerequisites for substantially higher TD/TC values, with the resulting nanocomposites still electronically insulating.

## 4. Polymer/BNNs Composites of Enhanced Cross-Plane or Isotropic TC

Since BNNs are close to planers, which tend to lie flat on the surface and orient horizontally, their uses as nano-fillers for polymer composites with high TC cross-planes or isotropics are intrinsically more challenging. The reported cross-plane TC values are often more than an order of magnitude lower than the in-plane TC values (Table 1 and Table 2). Nevertheless, there have been significant efforts made toward the fabrication and study of polymer/BNNs composites, driven by the needs for efficient cross-plane or isotropic thermal transport in many technological applications, including, for example, the demand for rapid heat dissipation in modern electronic devices [64,132]. Several approaches have been explored to construct 3D thermal transport networks with BNNs dispersed in the polymer matrix for their improved vertical alignment [133,134], including methods such as ice templating [64,130,132,135,136,137,138], magnetic and electric field-induced orientation [64,130,132,137,139], 3D printing, nano-filler surface modification with binders, and the interconnection of small BNs or other micro-nano structures with large-sized BNNs as additives [127,130,132,137].

**Figure 6:**
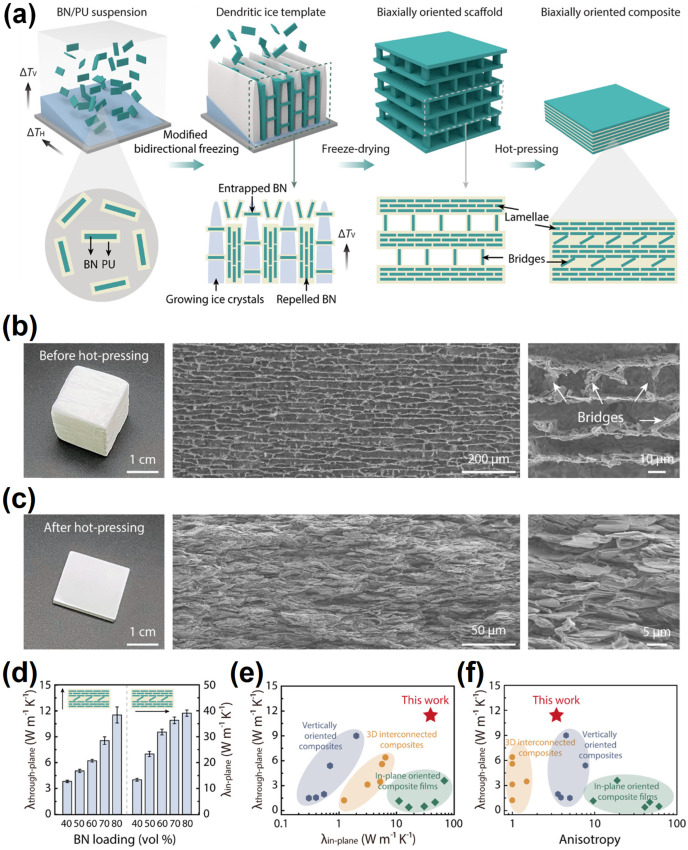
(**a**) Schematic diagram: Fabrication process of the BN/PU composites with a biaxially oriented network. (**b**) Structure and thermal property of BN/PU composites with a uniaxially oriented network. (**b**) Structure of the BN/PU porous scaffold (20 mm × 20 mm × 20 mm). The BN/PU porous scaffold shows a lamellar architecture with interlayer bridges. (**c**) Structure of the BN/PU bulk composite (20 mm × 20 mm × 2 mm). The lamellar structure was maintained after hot-pressing. (**d**) Through-plane and in-plane thermal conductivity of the BN/PU bulk composites at different BN loadings. (**e**,**f**) Comparison of thermal conductivity and anisotropy with other reported BN-based composite films and bulk composites. Reproduced/Adapted with permission from Ref. [140].

For ice templating [118,130,136,140], the basic idea is to prepare a porous structure with a regular pattern that is a replica of ice crystals, where the preparation is to freeze a specific aqueous colloidal suspension and then remove the ice crystals by sublimation and sintering. For example, Zhao, et al. [140] developed a method involving the use of a dendritic ice template to fabricate polyurethane/BN composites, aiming for a biaxially oriented thermal conductive network (Figure 6). In their experiment, an aqueous suspension of BNNs and polyurethane was poured into a plastic mold with a PDMS wedge, followed by freezing in liquid nitrogen and freeze-drying to obtain porous scaffolds. The scaffolds thus obtained were hot pressed along the direction perpendicular to the lamellar layers to generate a densified composite. The composite with 80 vol% BN loading was found to have an in-plane TC of 39.0 Wm^−1^K^−1^ and a cross-plane TC of ∼11.5 Wm^−1^K^−1^. In another study, Huang, et al. used an ice-templated assembly strategy for the purpose of creating networks of BNNs in polymer composites. They dispersed commercially acquired BN powder and BN nanocrystals (BNNCs) in isopropanol, followed by freeze-drying and then mixing with a polymer solution of poly(vinyl alcohol) in aqueous sodium alginate to form a gel. Monolith ice containing Ca^2+^ ions was pressed onto the gel, followed by solvent exchange and ambient drying. Measurements of the composites with 25 wt% BN loading showed that the maximum in-plane and cross-plane TC values could reach 20.3 Wm^−1^K^−1^ and 21.3 Wm^−1^K^−1^, respectively [142].

Magnetic and electric field-assisted alignments have found some success in manipulating the filler orientations in polymer/BNNs composites. For example, He, et al. coated BNNs with magnetic iron oxide nanoparticles to make the resulting nano-fillers magnetically responsive. Their incorporation into epoxy resin in the presence of an external magnetic field enabled the cross-plane alignment of the fillers in the composites. At 30 wt% BNNs loading, the cross-plane TC was as high as 14.55 Wm^−1^K^−1^ [147]. In a similar study, magnetically functionalized BNs were combined with polyvinyl pyrrolidone (PVP) for assembly into composites with a magnetic field-assisted slip casting method. The composites with BN loading of 62.6 vol% exhibited a cross-plane TC of 12.1 Wm^−1^K^−1^ [168]. There have been several other reports on the application of magnetic and electric alignment methods [139,169,170], including a more recent one by Li, et al. on using an electrostatic flocking method to align h-BN in epoxy composites [150]. The cross-plane TC performances were not so impressive in absolute values despite the relative improvements from those without the induced alignment.

The use of magnetic modification of BNNs to enable their alignment or controlled orientations in polymer composites may find some specific applications. Broadly speaking, however, the added issues and possibly complications due to the presence of the magnetic elements, which are essentially “impurities” in the composites for some other uses, might be limitations, and so could the associated higher costs.

The use of surface modified BNNs with binders has been another strategy for enhanced cross-plane TC performance. The strategy involves interconnecting nano-sized and micron-sized BN fillers or mixing fillers of different shapes and types with large-sized BNNs in the polymer composites, with additional aims such as improving interfacial compatibility for better phonon transmission between the fillers and the polymer matrix [48,143,144,145,146]. For example, Xu, et al. prepared silanol-modified BNNs by a combination of ultrasonic exfoliation and grafting with silanol groups for the fabrication of composites with polyamide 6/polyethylene terephthalate via solution mixing, rapid PA6 coagulation, and hot pressing [143]. The expectation, supported by simulation results, was that the modified BNNs were randomly orientated in the composites, thus more favorable to cross-plane thermal transport. The cross-plane TC value of the composite, 3.28 Wm^−1^K^−1^ at 55 wt% filler loading, represents a significant improvement from those of the composites with unmodified BNNs, but still not so impressive. More broadly, the critique on the magnetic alignment strategy above may also be applied to the approach of modified BNNs here, with opportunities for some specific needs but significant limitations in others.

**Figure 7:**
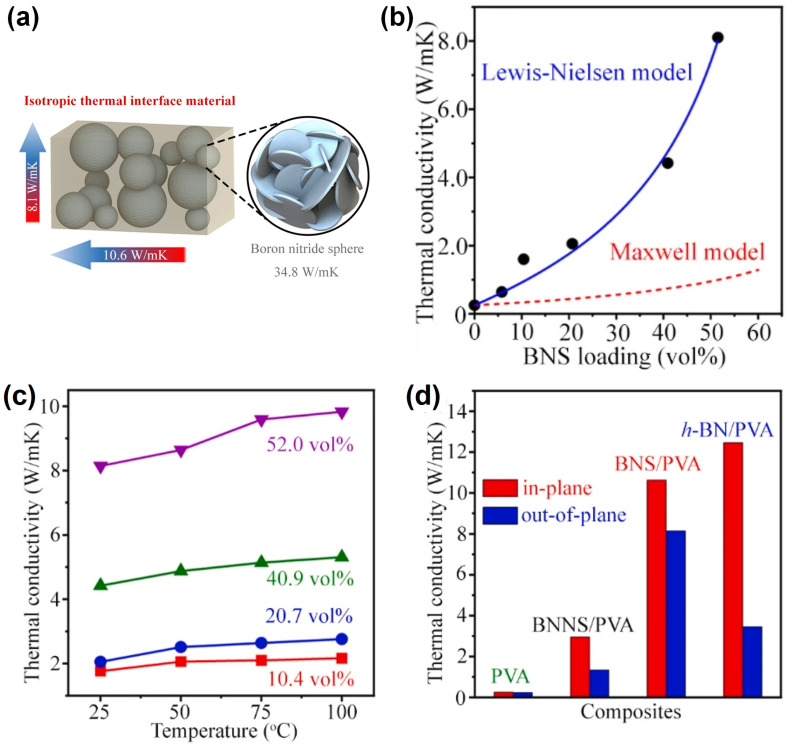
(**a**) Schematic diagram of fabricated isotropic thermal interface material. (**b**) Out-of-plane thermal conductivity of BNS/PVA as a function of BNSs loading (black dots), corresponding Lewis–Nielsen model fitting (blue curve), and Maxwell model simulation (red curve). (**c**) Out-of-plane thermal conductivity of BNS/PVA composites as a function of temperature. (**d**) Comparison of room temperature in-plane and out-of-plane thermal conductivities of four different composites. Reproduced/Adapted with permission from Ref. [144].

In a hybrid strategy, BNNs and BN nanoparticles (BNNPs) obtained both from ball milling under different conditions and dispersed in water were used to fabricate micron-sized BN spheres (BNSs) by spray drying and sintering at high temperature [144]. Here, high temperature sintering was designed to enhance the interactions between the BNNs in the presence of BNNPs and the TC of BNSs (Figure 7). The BNSs were then used as fillers for the PVA composites, which exhibited an in-plane TC value of 10.6 Wm^−1^K^−1^ and a cross-plane TC value of 8.1 Wm^−1^K^−1^ at 52 vol% loading of BNSs. Similarly, densified spherical h-BN microspheres were prepared by spray-drying and sintering from the precursor of an aqueous nano-BN suspension with Triton X-100 (Dow Chemical Company, Midland, MI, USA) and PVP and sodium silicate as binders. The microspheres were mixed with a pre-cured PDMS polymer and then cured using the hot-press molding method for the polymer composites. The composite with 60% content of the microspheres was pretty isotropic in TC values, around 5 Wm^−1^K^−1^ both in-plane and cross-plane [145].

There have been works conceptually from the further expansion of the hybrid approach, such as the one reported by Zuo, et al. [146]. They prepared hollow cube-like BN powder by using the precursor of cube NaCl as a template and boron oxide and ammonia as boron and nitrogen sources, respectively, for processing at a high temperature. The powder thus prepared was eight times lower in density than a typical BNNs sample. The incorporation of such BN powder into polyimide yielded composites of 4.93 Wm^−1^K^−1^ in cross-plane TC at 23.3 wt% BN loading. Yu, et al. [148] prepared one component by coating melamine–formaldehyde sponge with BNNs and another component by adding the dopamine-treated h-BN into epoxy matrix, and then impregnated the second component into the first, followed by deformation, crosslinking, molding, and compression for the targeted polymer composites. At 35.9 wt% BN loading, the composites exhibited in-plane and cross-plane TC values of 10.20 Wm^−1^K^−1^ and 4.95 Wm^−1^K^−1^, respectively.

There was also a report on using composite particles of zinc oxide and BNNs as fillers in polymer composites, although their in-plane and cross-plane TC values were pretty low [153]. More recently, Jia, et al. introduced liquid metal as a bonding agent and thermal linker in composites with BN platelets, thus taking advantage of the high TC and excellent fluidity of liquid metal. The liquid metal/BN powders were prepared by mechanical agitation and compression, and then converted to self-standing bulks, namely special composites of liquid metal with BN. At a 10% volume fraction of liquid metal, the composite could reach maximum in-plane and cross-plane TC values of 82.2 Wm^−1^K^−1^ and 20.6 Wm^−1^K^−1^, respectively [141], obviously higher than those of most polymer/BNNs composites. However, the use of electrically conductive liquid metal to replace the polymer matrix in composites defeats the purpose of using BNNs as nano-fillers of high TC but no EC. In fact, even higher thermal transport performance can be achieved with polymer composites with fewer-layer graphene (GNs) fillers or high-quality films of neat GNs. Thus, the special composites of liquid metal with BN may only make sense in some very special cases.

**Figure 8:**
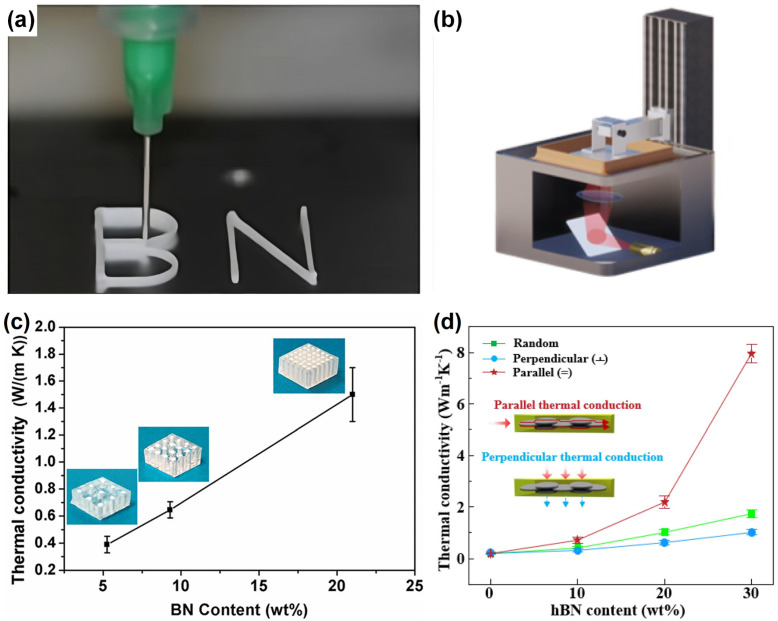
(**a**) Optical image of the structure being printed using the Direct Ink Writing 3D printing method. (**b**) Schematic diagram of the Digital Light Processing 3D printing process. (**c**) Through-plane thermal conductivity of BN array/PDMS materials made with three-by-three, four-by-four, and six-by-six BN rods, which corresponds to BN loadings of 5.3, 9.3, and 21.0 wt%, respectively. (**d**) Thermal conductivity of the DCR/h-BN composites. Reproduced/Adapted with permission from Refs. [152,171].

In recent years, the additive manufacturing tool 3D printing has been employed for rapid and/or more controllable fabrication of customized polymer composites or their derived architectures with enhanced TC performance. Typically, the melt extrusion and shear force within the nozzle used in the 3D printing process could orient BNs in the polymer matrix along the printing direction, thus providing a new alignment approach. For example, Liang, et al. reported the 3D printing of BN/Pluronic^®^ F-127 ink-filament (BASF, Ludwigshafen, Germany) in which the densely packed BNNs were printed along the vertical direction through the generation of a strong shear-force during the extrusion of the ink filaments from the fine nozzle [171]. The as-printed array of BNNs was then encapsulated in polydimethylsiloxane for a somewhat more thermally conductive composite than the neat polymer and the composite without the alignment (Figure 8a,c). More recently, Wang, et al. used digital light processing 3D printing to orient h-BNs in the mixture of epoxy resin and UV curable resin [152]. The resulting thin-layer (15 microns) composite of 30 wt% h-BN loading exhibited a respectable in-plane TC of 7.962 Wm^−1^K^−1^ but a much lower cross-plane TC, as shown in Figure 8b,d. In an effort to combine 3D printing with the use of magnetically modified BN, Lee, et al. fabricated a vertically aligned composite of iron oxide-decorated BN in UV curable resin by using magnetic field-assisted 3D printing [172], although the observed TC value was only 1.36 Wm^−1^K^−1^ with a significant filler content (17.5 vol%) in the composite. It should be pointed out that despite the limited success of 3D printing in the fabrication of polymer composites for a much enhanced cross-plane TC performance, a bright future for this extremely powerful and highly versatile additive manufacturing tool for this purpose can be envisaged.

The direct comparison of the thermal conductivity performance of BN/polymer nanocomposites from these methods is challenging due to variations in processing parameters, BN exfoliation, BN fractions, and polymers. Although these approaches have shown their potential to slightly improve the cross-plane thermal conductivity of BN/polymer nanocomposites, they still face significant limitations that deserve further investigations. For example, the sacrificial template and magnetic and electric field-induced orientation methods require multiple complex steps, considerable energy input, and often produce composites with a short-range order and orientation, poor mechanical strength, poor bonding and interfacial compatibility, and poor control over the film thickness and BNNs distribution, making them still not compatible for batch processing and production. The non-uniform doping and tendency to agglomeration of magnetic and other metal nanoparticles on the surface of BNNs significantly affects the electric, mechanical, and other properties of BNNs, leading to inconsistencies in nanocomposite performance and effectively limiting the performance of electronic devices. The applied electric field also requires the use of an ultra-high voltage due to the high viscosities and the breakdown voltage of the polymer, resulting in the production of only small-scale aligned films. On the other hand, the interconnection of compatible nano-or micron-sized BN structures with large BNNs and 3D printing and their combination seem to be more promising methods, with significant advantages, including the fast fabrication of customized three-dimensional products with a complex geometry and high resolution approaching the nanoscale, better interconnection and orientation of BN structures, better distribution of BNNs within polymer composites, a wide selection of materials, and reduced waste.

## 5. Summary and Perspectives

The use of BNNs in polymer composites of high TC for thermal management applications has clearly attracted more attention, likely due to the increasing needs for and the recognition of the electrically insulating nature of the composites that are uniquely suited or have major advantages for uses in electrical and electronic devices and systems. There has been meaningful progress made over the last few years, mostly incremental without any major breakthroughs, which present both challenges and opportunities, especially in using BNs for composites of high isotropic TC performance.

There are some critical needs for consensuses in the relevant research field that are necessary for healthy advances of the field, among which a clear distinction between the mechanisms of TC and EC is especially important and consequential. In composite materials, TC is due to phonon transport, a term popular in physics, or with a simple translation, to its association with vibrations in the materials, which is clearly different from electron transport via the percolation network for EC. Vibrations are materials properties that are volume and mass dependent, so just to have the percolation network constructed with a small amount of fillers is insufficient for any significant thermal transport in the composites. Evidently, in composites of nano-fillers that are high in both TC and EC, there is a major divergence in the dependencies of TC and EC performances on filler loadings. In a representative example, when carbon nanotubes are used as fillers in composites at high loadings or even as neat mats, the divergence between the observed TC and EC performances is rather extreme. In fact, it is generally more challenging to achieve high TC performance with composites containing high TC nano-fillers, but at the same time, it also leaves a lot more room for the manipulation of nano-filler structures and morphologies and their dispersions in the composites to achieve high TC performance.

Another consensus needed is on the systematic calibration of the various TC/TD measurement methods, both absolutely in individual methods against well-established standards and relatively across different methods for the necessary comparability among their generated results. For example, some of the instruments originally designed for primarily cross-plane TD measurements, as made clear by the available instrument design principles and blueprints and also the disclaimer of the manufacturer, are subject to intrinsic limitations when employed for in-plane TD measurements.

For polymer/BNNs composites of the desired high TC performance, the “high quality” of BNNs is obviously very important yet seemingly remains an elusive concept. Among the proposed more desirable BNNs have been those of certain characteristics, such as high crystallinity, fewer defects, larger lateral size, uniformly thinner, right orientation, and/or dispersibility and interfacial compatibility with the polymer matrix, among some others. However, extreme challenges remain for the necessary critical evaluations of the characteristics in comparable composites under comparable conditions. The possibility or even likelihood for the in-plane and cross-plane TC performances to favor different sets of characteristics of the nano-fillers would add more complications.

Many of the technological applications require or prefer composite materials of high isotropic or cross-plane TC performance. As planar nano-fillers, BNNs are naturally more favorable to in-plane thermal transport in polymeric composites. In recent investigations, a number of processing strategies were employed to orient BNNs in composites for enhancing the cross-plane TC performance, as highlighted in the relevant section above. Particularly interesting is the emerging use of 3D printing or its combination with another processing tool for the fabrication of polymer/BNNs composites in which the fillers are selectively aligned. Going forward, the additive manufacturing strategies are very promising, and more 3D printed composites and devices of high thermal transport performances in specifically selected orientations may be envisaged.

## Figures and Tables

**Table 1:** In-plane and Cross-plane TC Values for Polymeric Nanocomposite Films with BNNs from Different Exfoliation Methods/Conditions Targeting Anisotropic Configuration.

Polymer *^a^*	Type of Fillers *^b^*	Exfoliation and Fillers Synthesis Method	Composites Fabrication Technique	Loading	In-Plane TC (Wm^−1^K^−1^) *^c^*	Cross-Plane TC (Wm^−1^K^−1^) *^c^*	Method *^d^*	Ref.
TOCNF/PDA ^(1)^	BNNs	Ball milling	Vacuum filtration and hot pressing	50 wt%	23.49	2.25	LFA	[91]
Cellulose	BNNs	Sonication and ball milling	Gelation, stretching, drying	50 wt%	20.41	0.69	LFA-467	[92]
PVA/Chitosan	BNNs	sonication	Casting	20 wt%	**38.21**	–	LFA-467	[93]
PBO/PDA ^(2)^	BNNs	Ball milling	Hot pressing	37.5 wt%	**45.15**	0.772	LFA-467	[94]
PPGTD, IPDI and AD	BNNs	Sonication	Hot pressing	38 wt%	12.62	0.63	DXF-900	[95]
CCNF	BNNs	Sonication	Casting	50 wt%	17.31	–	LFA-467	[96]
PMMA/PN	BNNs	Ball milling	Hot pressing	40 vol%	7.835	1.622	Hot Disk	[97]
EP	BNNs@IL	Ball milling	Casting	45 wt%	8.3	0.8	LFA-467	[98]
Chitosan	BNNs	Sonication and ball milling	Gelation, stretching, drying	8 wt%	5.59	–	LFA-467	[99]
P(VDF-HFP)	BNNs	Sonication	Blade casting, folding, and hot pressing	25 vol%	7.26	–	LFA-467	[100]
PVA	BNNs	Ball milling	Vacuum-assisted filtration	90 wt%	27.3	–	LFA-467	[101]
PVA	BNNs	Microfluidization	Vacuum-assisted filtration	83 wt%	67.6	–	LFA-467	[102]
PVA/PDA ^(2)^	BNNs	Sonication	Electrospinning and vacuum filtration	35.5 wt%	16.6	–	LFA-467	[103]
TPU	BNNs	Sonication	Spray coating and hot pressing.	50 wt%	20.65	5.77	LFA-467	[104]
PBO	BNNs	Sonication	Sol–gel-film conversion method	10 wt%	**21.34**	–	LFA-467	[105]
ANF	BNNs	Sonication	Mechanical agitation, ultrasonication and filtration	70 wt%	28.75	–	LFA-467	[106]
CNF	BNNs	Sonication	vacuum filtration.	33.3 wt%	13.46	–	LFA-447	[107]
PP	BNNs	Sonication	Hot pressing	19.1 vol%	4.08	–	LFA-467	[108]
PVA	BNNs	Ball milling	Scraping method	30 wt%	11.5	–	LFA-467	[109]
PAN	BNNs	Ball milling	Electrospinning and electrospraying	40 wt%	**24.98**	–	LFA-447	[110]
PI	BNNs	Hydrothermal	Electrospinning and electrospraying	50 wt%	7.58	–	LFA-467	[111]
PS	BNNs	Sonication	Solution mixing	10 wt%	1.6	–	LFA-467	[112]
ANF	BNNs@Ag NW	Solvothermal and in situ growth	Hot pressing	50 wt%	9.44	0.75	Hot Disk	[113]
**Other Selected In-plane and Cross-plane TC Values from Different Methods/Conditions Before 2021**								
PI/PAA	BNNs	Sonication	Casting	30 wt%	2.38	1.14	LFA-467	[114]
PVA	h-BN	Sonication	Solution mixing	10 wt%	6.43	0.34	LFA-447	[115]
PVDF	BNNs	Liquid-phase exfoliation	Electrospinning	33 wt%	16.3	–	LFA-467	[116]
PVA	BNNs@Ag NW	Ball milling	Electrospinning	33 wt%	10.9	–	LFA-467	[117]
EP	BNNs	Ball milling	Ice templating and infiltration	34 vol%	4.42	–	LFA-467	[118]
Natural rubber/PDA ^(2)^	h-BN		Rolling and shearing	30 vol%	0.39	–	DXF 500	[119]
PVA	BNNs	Sonication	Electrospinning and pressing vacuum filtration	22 vol%	**21.4**	–	LFA-467	[120]
TPU	h-BN	Ball milling	Precipitation	95 wt%	50.3	6.9	LFA-447	[121]
PVDF	BNNs	Ball milling	Solution mixing	60 wt%	11.88	–	LFA-447	[122]
PVA	BNNs	Ball milling	Electrospinning	40 wt%	19.9	–	LFA-467	[123]
PS/PP	BNNs		Solution mixing and hot pressing	50 wt%	5.57	–	Hot Disk	[124]
PVA	BNNs	Sonication	Casting and stretching	15 vol%	**13**	–	LaserPIT	[17]
PVA	BNNs	Reflux	Casting	20 vol%	**12**	–	LaserPIT	[58]
PVA	BNNs	Ball milling	Casting	20 vol%	8.2	–	LaserPIT	[58]
PVA	BNNs	Hydrothermal	Casting	20 vol%	16	–	LaserPIT	[58]
PVA	BNNs	Hydrothermal	Casting	40 vol%	**26**	–	LaserPIT	[125]
PE	BNNs	Sonication	Cross-linking and casting	40 wt%	**41**	–	LaserPIT	[126]

*^a^* TOCNF: TEMPO-oxidized nanocellulose fibers; ^(1)^ PDA: polydopamine hydrochloride; PVA: poly(vinyl alcohol); PBO: poly-p-phenylene benzobisoxazole; ^(2)^ PDA: polydopamine; PPGTD: poly (propylene glycol) with tolylene 2,4-diisocyanate; IPDI: isophorone diisocyanate; AD: 4-aminophenyl disulfide; CCNF: carboxymethylated cellulose nanofibrils; PMMA: poly(methyl methacrylate); PN: phthalonitrile; EP: epoxy; P(VDF-HFP): poly(vinylidene fluoride hexafluoropropylene); ANF: aramid nanofiber; PP: polypropylene; PAA: polyacrylic acid; PI: polyimide; PVDF: polyvinylidene fluoride; EP: epoxy; PS: polystyrene; TPU: polyurethane. *^b^* IL: ionic liquid; Ag NW: silver nanowires. *^c^* Benchmark in-plane TC values are marked in bold. Usually, the in-plane TC values of BNNs/polymer composites are 2 to 3 orders of magnitude higher than that of bulk polymers. *^d^* LaserPIT: Designed specifically for in-plane TD; LFA-447 and LFA-467: Based on laser flash method; DXF-500 and DXF-900: Based on xenon light flash; Steady-State: Measuring the temperature difference due to steady-state heat; TA3: Thermo-wave analysis with periodic laser heating; Hot Disk: A metal disk as a heat source and for sensing; TDTR: Based on ultrashort laser pulses and pump-probe detections.

**Table 2:** In-plane and Cross-plane TC Values for Polymeric Nanocomposite Films with BNNs from Different Exfoliation Methods/Conditions Targeting Isotropic Configuration.

Polymer *^a^*	Type of Fillers *^b^*	Exfoliation Method	Composites Fabrication Technique	Loading	In-Plane TC (Wm^−1^K^−1^) *^c^*	Cross-Plane TC (Wm^−1^K^−1^) *^c^*	Method *^d^*	Ref.
PU	BNNs	Ball milling	Ice templating and hot pressing	80 vol%	39	11.5	Hot Disk	[140]
LM	BN Platelets	–	Mechanical agitation	90/10 vol% BN/LM	82.2	20.6	Hot Disk	[141]
PVA	h-BN/BNNC	Freeze-drying	Ice pressing	25 wt%	**20.3**	**21.3**	LFA-467	[142]
PA6/PET	BNNs	Sonication	Solution mixing, solidification, and hot pressing	55 wt%	–	3.28	Hot Disk	[143]
PVA	BNNs/BNNP	Ball-milling	Vacuum filtration	52 vol%	10.6	8.1	LFA-457	[144]
PDMS	h-BN	Sonication, spray drying and sintering method	Hot pressing	60 vol%	5.6	4.3	LFA-447	[145]
PAA	BNNs@NaCl	Vacuum filtration	Vacuum defoaming	23.3 wt%	–	4.93	LFA-447	[146]
EP	BNNs	Sonication	Solution mixing	30 wt%	–	**14.55**	TDTR	[147]
EP/PDA	BNNs	Sonication	Solution mixing	35.9 wt%	10.20	4.95	Laser flash	[148]
EP	h-BN	–	Hot pressing	50 wt%	–	4.27	LFA-467	[149]
EP	h-BN	–	Electrostatic flocking	17.6 wt%	–	0.65	Hot Disk	[150]
EP	BNNs/Ag NWs@Ni	Ball-milling	Hot pressing	40 wt% BN/Ag NWs@Ni	0.723	0.824	LFA-467	[151]
EP	h-BN	–	3D printing	30 wt%	7.962	0.893	LFA-467	[152]
PMDA	BNNs	Liquid-phase exfoliation	Vacuum defoaming	30 wt% ZnO@BN	2.235	0.853	LFA-467	[153]
PAA	GF-BNNs	–	Magnetic field induction	30 wt%	2.532	0.425	Hot Disk	[154]
EP	BNNs	Sonication	Vacuum filtration	15.4 vol%	–	2.01	LFA-467	[155]
**Other Selected In-plane and Cross-plane TC Values from Different Methods/Conditions Before 2021**								
EP	BNNs	Freezing	Ice templating and infiltration	9.29 vol%	2.85	2.40	LFA-467	[138]
EP	h-BN	Sonication	Vacuum filtration	44 vol%	–	**9**	LFA-447	[156]
EP	h-BN/Ag NP	Sonication	Hot pressing	62.2 wt%	23.1	3.6	LFA-467	[157]
Silicone rubber	h-BN	–	Hot pressing	39.8 vol%	–	5.4	LFA-427	[158]
PVA	h-BN	Sonication	Vacuum filtration	27 vol%	8.44	1.63	LFA-447	[159]
EP	BNNs	Sonication	Solution Mixing	50 vol%	–	**9.81**	LFA-447	[160]
PDMS/PVA	BNNs	Liquid-phase exfoliation	Electrospinning and vacuum-assisted impregnation	50 vol%	–	1.94	LFA-467	[133]
EP	BNNs	Sonication	Vacuum infiltration	4.4 vol%	–	1.56	LFA-447	[161]
Silicone gel	h-BN	–	Solution Mixing	9.14 vol%	–	0.357	LFA-457	[162]
PE	h-BN	–	Melt blending	5.97 vol%	–	1.37	LFA-467	[163]
PMMA	BNNs	Sonication	Vacuum infiltration and wet process	80 wt%	14.7	10.2	LFA-447	[164]
EP	BNNs	Liquid-phase exfoliation	Vacuum filtration and impregnation	21.9 vol%	1.43	4.22	Hot Disk	[165]
PTFE	h-BN/AlN	–	Compression molding	30 vol%	–	1.04	LFA-447	[166]
PCL	h-BN	Ice templating	In situ polymerization	13.41 wt%	1.42	1.01	LFA-467	[167]

*^a^* PU: polyurethane; PVA: poly(vinyl alcohol); PA6: polyamide 6; PET: polyethylene terephthalate; PDMS: *polydimethylsiloxane*; PAA: polyacrylic acid; EP: epoxy; PDA: polydopamine; PMDA: phthalic anhydride; PE: polyethylene; PVDF: polyvinylidene fluoride; PMMA: poly(methyl methacrylate); PTFE: polytetrafluoroethylene; PCL: polycaprolactone. *^b^* LM: liquid metal; BNNCs: BN nanocrystals; IL: ionic liquid; AgNW: silver nanowires; Ag NP: silver nanoparticles; GF: hybrid nanoparticle of reduced graphene oxide and ferro-oxide; AlN: aluminum nitride. *^c^* Benchmark cross-plane and in-plane TC values are marked in bold. *^d^* LaserPIT: Designed specifically for in-plane TD; LFA-427, LFA-447, LFA-457, and LFA-467: Based on laser flash method; Steady-State: Measuring the temperature difference due to steady-state heat; TA3: Thermo-wave analysis with periodic laser heating; Hot Disk: A metal disk as the heat source and for sensing; TDTR: Based on ultrashort laser pulses and pump-probe detections.

## Data Availability

No applicable.
